# Relationship between Anaemia, Haemolysis, Inflammation and Haem Oxygenase-1 at Admission with Sepsis: a pilot study

**DOI:** 10.1038/s41598-018-29558-5

**Published:** 2018-07-25

**Authors:** Phebe Ekregbesi, Manu Shankar-Hari, Christian Bottomley, Eleanor M. Riley, Jason P. Mooney

**Affiliations:** 1 Department of Immunology and Infection, London School of Hygiene and Tropical Medicine, London, United Kingdom; 2 Department of Critical Care Medicine, Guy’s and St Thomas’ NHS Foundation Trust, London, United Kingdom; 3 Department of Infectious Disease Epidemiology, London School of Hygiene and Tropical Medicine, London, United Kingdom; 4 Division of Infection and Immunity, The Roslin Institute, University of Edinburgh, Edinburgh, United Kingdom

## Abstract

Upregulation of haem oxygenase-1 (HO-1), due to haemolysis and/or inflammation, can lead to impaired immune function. Anaemia is common among sepsis patients, but the consequences of sepsis-associated anaemia are poorly understood. Here, our objective was to determine the prevalence and extent of anaemia, haemolysis, inflammation, and HO-1 induction after early hospital admission. We hypothesised that inflammation- or infection-induced haemolysis contributes to sepsis-associated anaemia and that this will lead to expression of HO-1. In this study, plasma obtained from seventy adult patients within 12 hours of admission to intensive care due to sepsis were analysed for anaemia, haemolysis and inflammatory markers by ELISA and microbead array. The majority (82.6%) of patients were anaemic with evidence of haemolysis (raised haem, haptoglobin, haemopexin, and HO-1 concentrations). Interestingly, concentrations of both haemoglobin and IL-10 were moderately positively correlated with HO-1 concentration (Hb: r = 0.32, p = 0.007; IL-10 r = 0.39, p = 0.0008) whereas HO-1 concentration was weakly negatively correlated with haemopexin (r = −0.23, p = 0.055). Anaemia, while common, was not associated with HO-1 concentration. After adjusting for confounding, HO-1 induction appears to be associated primarily with IL-10 concentration rather than haemolysis. Disease severity at diagnosis was correlated with early plasma IL-10 (r = 0.35, p = 0.003) and HO-1 (r = 0.24, p = 0.048) concentrations. Notably, admission levels of haem, HO-1, and IL-10 were indicators of survival.

## Introduction

Sepsis is the dysregulated host response to infection leading to life-threatening organ dysfunction^1,2^. In the UK, there are approximately 147,000 cases of sepsis per year with an estimated 30% mortality; survivors face lifelong complications^3–5^. Anaemia is highly prevalent among intensive care patients^6,7^ and can be common among the elderly^8^ who are at particular risk of sepsis. Anaemia in septic patients is multifactorial^9–11^, it may reflect anaemia of chronic disease (ACD), haemolysis, repeated phlebotomy and haemodilution. ACD is an immune driven distortion of iron homeostasis, red cell production and red cell lifespan driven by interleukin (IL)-6 and hepcidin^12–14^ whereas haemolysis may result from the action of bacterial haemolysins, immune-mediated erythrocyte destruction or oxidative damage. The causes and consequences of anaemia in sepsis-related critical illness have not been fully explored.

Haemolysis leads to the liberation of haemoglobin; haemoglobin catabolism produces haem^15^ which is a highly cytotoxic pro-oxidant. At steady state, the host scavenger protein haptoglobin binds haemoglobin; the complex is taken up by scavenger cells and the haem is catabolized by the constitutively produced enzyme, haem oxygenase (HO)-2. When this homeostatic process is overwhelmed, free haem is scavenged and neutralized by haemopexin. After cellular internalisation of haem-haemopexin complexes by CD91, haem is degraded to carbon monoxide, iron, and biliverdin by the inducible isoform of haem oxygenase, HO-1^16^.

HO-1 induction has been reported in liver and in blood monocytes of sepsis patients^17,18^. In humans with malaria-induced haemolysis, raised plasma HO-1 concentrations correlate with severely impaired neutrophil respiratory burst^19,20^ and, in mice with haemolysis, neutrophil function can be restored by specific inhibition of HO-1 activity^19^ suggesting a role for HO-1 in increased susceptibility to invasive bacterial disease^19,21^. However, HO-1 concentrations also correlate with the acute phase response (C reactive protein)^19^ indicating a role for host inflammatory responses in initiating or potentiating HO-1 induction. These synergistic interactions between anaemia, infection, inflammation and HO-1 may be occurring in sepsis patients. For example, in human sepsis, haemoglobin concentrations decrease in a hepcidin-dependent manner during admission, hepcidin and IL-6 concentrations are positively correlated and hepcidin concentrations are highest in those with the most severe disease^22^. Moreover, free haemoglobin is associated with reduced survival in sepsis^23^ and higher concentrations of haptoglobin and haemopexin have been observed in survivors than among those who died, indicating that haemolysis may be occurring in sepsis, contributing to anaemia^16,24^.

In this context, we hypothesized that haemolysis may be contributing to anaemia in critically ill sepsis patients and may result in induction of HO-1, which may synergize with inflammation to impair innate immune function. In this pilot study, hospital records and plasma were analysed from samples collected within 12 hours of ICU admission of sepsis patients whom did not have any documented immune comorbidity^25^. The aims of the study were to determine the prevalence of, and the interaction between, anaemia, haemolysis, inflammation and disease severity and particularly, to understand its relationship to HO-1 induction at the time of admission to the ICU for bacterial sepsis. These data provide a secure foundation for future prospective studies of the relationship between sepsis, haemolysis and immune function.

## Results

### **C**haracteristics of sepsis cohort at admission

For 70 patients at admission with sepsis^25^, median age was 64 years (range 18–89), and 62.9% were male. The respiratory tract was the most common infection site (65.7%), followed by wound and soft tissue (12.9%), intra-abdominal (11.4%), bladder (8.6%) and bone (1.4%). The median APACHE II score was 18.5 (range 8–37) and the median SOFA score was 7.0 (range 3–16). An acute hospital mortality of 27.1% was comparable to previous reports from England^26,27^. These clinical characteristics are summarized in Table S1.

Temperature, circulating leucocyte counts and inflammatory markers among sepsis patients were compared to the healthy reference range for the hospital (Fig. S1, Table S2). Twenty-three patients (33%) were febrile (>38 °C) and 8 (11%) were hypothermic (<35 °C) (Fig. S1A). Median platelet counts were low in sepsis patients and 20 patients (29%) were clinically thrombocytopenic (<150,000/μL) (Fig. S1B). Median lymphocyte counts were below reference levels (Fig. S1C) whereas neutrophil counts and C-reactive protein (CRP) concentrations were above normal (Fig. S1D,E).

### Sepsis patients are mildly to moderately anaemic at admission

**Figure 1:**
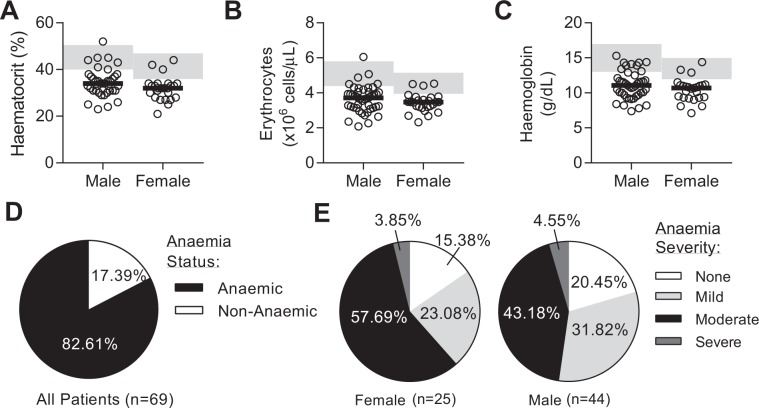
The majority of patients with sepsis have anaemia. Clinical parameters upon admission to the intensive care unit (ICU) after sepsis diagnosis. Erythrocyte parameters shown by gender for (**A**) haematocrit, (**B**) erythrocyte counts, and (**C**) haemoglobin. Pie charts showing proportion of patients with (**D**) anaemia (determined by haemoglobin concentration), and (**H**) stratified by gender and classification of severity using WHO recommendations (males: non-anaemic (>13 g/dL), mild (11–12.9 g/dL), moderate (8–10.9 g/dL), and severe (<8 g/dL). For females: non-anaemic (>12 g/dL), mild (11–11.9 g/dL), moderate (8–10.9 g/dL), and severe (<8 g/dL). Data collected from complete blood count (CBC) data. Dot plots show individual patient parameters. Black lines represent medians. Shaded areas represent healthy reference ranges (see Table S2). Sepsis patients, n = 70; haemoglobin data for 1 patient was missing.

**Figure 2:**
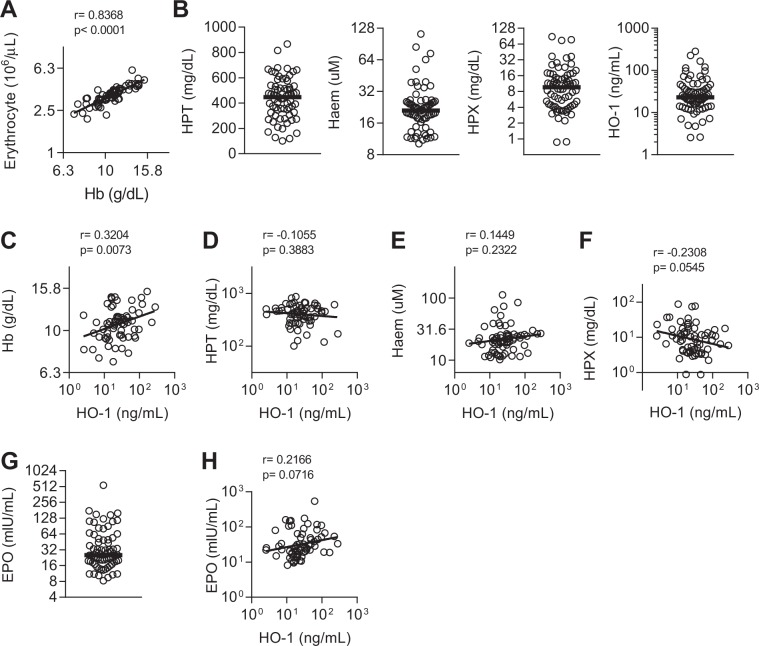
Haemolysis and HO-1 induction in sepsis patients. (**A**) Correlation between haemoglobin (Hb) concentration and circulating erythrocytes. (**B**) Plasma concentration of haptoglobin (HPT), haem, haemopexin (HPX), or haem oxygenase-1 (HO-1), and (**G**) erythropoietin (EPO). Dot plots show individual patient parameters. Black lines represent medians of septic patients, n = 70. Correlations between plasma HO-1 and (**C**) Hb, (**D**) HPT, (**E**) haem, (**F**) HPX, and (**H**) EPO. Log-transformed data shown with linear regression line. Pearson r and p-value shown.

Median haematocrit, red cell count and haemoglobin concentration were all below the healthy range in both male and female sepsis patients (Fig. 1A–C, Table S2). According to WHO guidelines^28^, 83% of patients were clinically anaemic at admission (Hb < 13 g/dL in males, Hb < 12 g/dL in females) (Fig. 1D), with most being moderately anaemic, i.e., with haemoglobin concentrations in the range 8–10.9 g/dL (Fig. 1E). Total haemoglobin (Hb) concentration was highly correlated with erythrocyte count (r = 0.84, p < 0.0001) (Fig. 2A). Mean corpuscular volume (MCV) was within the normal range for most patients (Fig. S1F).

### Sepsis and HO-1

As HO-1 can be induced by haemolysis and, independently, by cytokines (in particular, interleukin (IL)-10^29–32^), we next determined the levels of plasma HO-1 and whether haemolysis (defined by haem and haemopexin levels) or IL-10 was the most likely driver of HO-1 induction.

### HO-1 induction in sepsis patients correlates with haemolysis and haem scavenging

We measured plasma haem, haptoglobin (HPT), haemopexin (HPX) and HO-1 concentrations (Fig. 2B), as indicators of haemolysis. Haem concentrations (median 21.1 uM) were markedly higher than reported for healthy individuals (0.2 uM^33^) and haemopexin concentrations (median 9.6 mg/dL) were markedly lower (77 mg/dL^34^). HO-1 concentration was weakly negatively correlated with the haem scavenger protein, HPX (r = −0.23, p = 0.05) (Fig. 2F). The median erythropoietin (EPO) concentration was above the normal range (2–20 mIU/ml) (Fig. 2G). Therefore, while sepsis may be associated with haemolysis, only those haemolytic markers associated with haem scavenging (representing the total free-haem pool) correlate with HO-1 induction.

### Relationship between HO-1 induction and cytokine concentrations in sepsis patients

**Figure 3:**
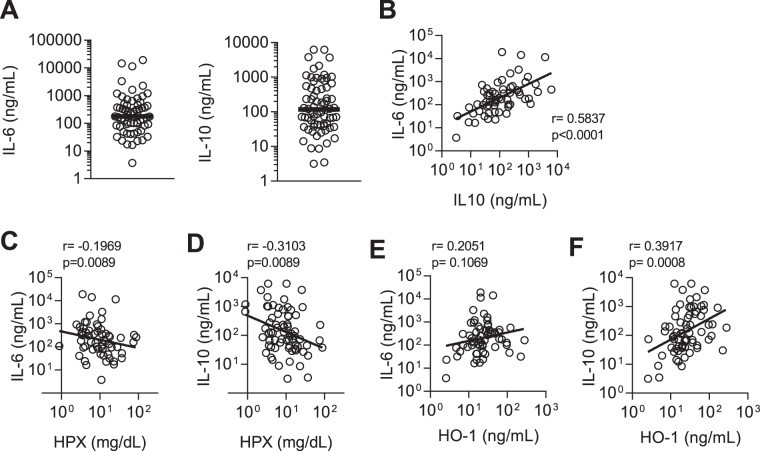
Cytokines and their relationship to haemolysis and HO-1 in sepsis patients. Plasma concentration of (**A**) interleukin (IL)-6 and IL-10 for sepsis patients; n = 70 in all cases except n = 63 for IL-6 (7 values exceeded the measurable range). Dot plots show individual patient parameters. Black lines represent medians of sepsis patients. Correlations for the relationship between IL-10 and IL-6 (**B**), and between IL-6 and IL-10 and haemopexin (HPX) (**C**,**D**), and between IL-6 and IL-10 and haem oxygenase-1 (HO-1) **(E**,**F)**. Log-transformed data shown with linear regression line. Pearson r and p-value shown.

As HO-1-mediated catabolism of haem to carbon monoxide is a major pathway for the anti-inflammatory properties of IL-10^29^, we measured concentrations of IL-10, tumour necrosis factor alpha (TNFα), granulocyte-colony stimulating factor (G-CSF), and IL-6 (Figs 3A–D and S2). IL-6 was significantly (positively) correlated with IL-10 concentration (r = 0.58, p < 0.0001) (Fig. 3B). None of the cytokines measured correlated with either anaemia (Hb) or haem concentrations (Fig. S3), but all showed significant, albeit weak, inverse correlations with haemopexin concentration (Figs 3C,D and S2). IL-10 was the only cytokine to be moderately and significantly correlated with HO-1 concentration (r = 0.39, p = 0.0008) (Figs 3E,F and S2). After adjusting for haemolysis (defined by both haem and HPX concentrations), IL-10 remained significantly associated with HO-1 (Table 1) whereas after adjusting for IL-10 concentration, neither haem nor HPX became or remained significantly associated with HO-1. Thus, whilst haemolysis (as measured by low HPX, reflecting cumulative exposure to haem) and IL-10 are both associated with markers of inflammation (IL-6, TNFα, and G-CSF), IL-10 is most closely associated with raised HO-1 in sepsis patients at the time of admission.

**Table 1:** Crude and adjusted correlations.

Variables	Correlation	p-value	Partial Correlation	p-value
IL-10 vs HO-1*	0.392	<0.001	0.326	0.006
Haem vs HO-1^†^	0.145	0.232	0.07	0.571
HPX vs HO-1^†^	−0.231	0.05	−0.125	0.303
IL-10 vs APACHE II^§^	0.349	0.003	0.287	0.016
HO-1 vs APACHE II^†^	0.237	0.048	0.117	0.337

Pearson’s correlation and p-value calculated using log-transformed data. *partial correlation adjusted for haem and HPX, ^†^partial correlation adjusted for IL-10, ^§^partial correlation adjusted for HO-1.

### Elevated IL-10 and HO-1 are associated with disease severity and mortality in sepsis

**Figure 4:**
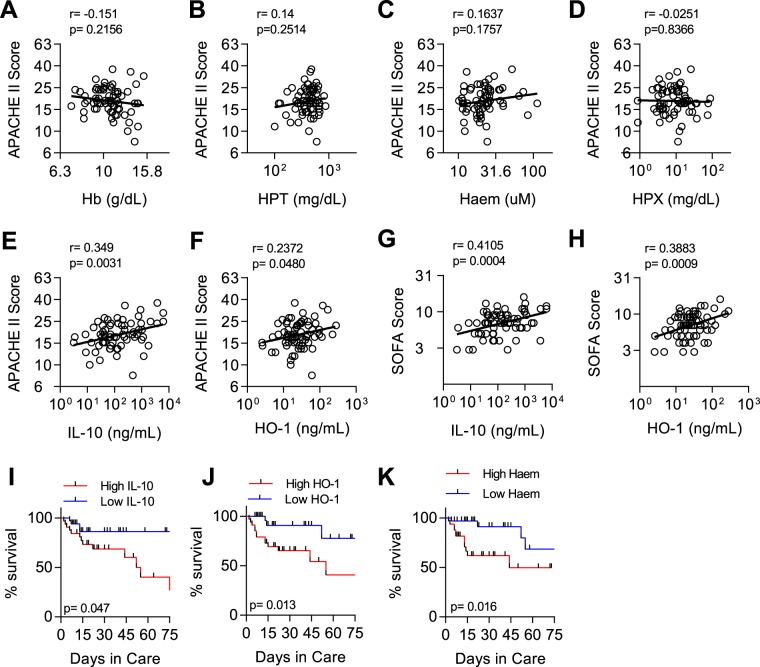
Elevated IL-10 and HO-1 are associated with severity of sepsis. Correlations for the relationship between APACHE II score and (**A**) Haemoglobin (Hb), (**B**) haptoglobin (HPT), **(C)** haem, (**D**) haemopexin (HPX), (**E**) IL-10, and (**F**) HO-1. Correlations for the relationship between SOFA score and (**G**) IL-10, and (**H**) HO-1. Log-transformed data shown with linear regression line. Pearson r and p-value shown. Kaplan–Meier survival curves with hazard ratio comparing individuals above the median to those below the median for (**I**) IL-10 (median 117.89 ng/mL), (**J**) HO-1 (median 23.105 ng/mL), and (**K**) haem (median 21.075 uM).

HO-1 can be induced by haemolysis and (in response to inflammation) by IL-10 and simultaneously increases tolerance (resilience) to infection (by detoxifying haem and producing tissue-protective carbon monoxide)^35,36^ whilst reducing resistance to infection by impairing immune function^19,37^. However, inflammation, and its subsequent regulation by IL-10, is a strong predictor of mortality in sepsis^38^ and IL-10 concentration is highly correlated with disease severity as measured, for example, by the APACHE II score^39–41^, suggesting that the immune suppressive effects of HO-1 may outweigh its tissue protective effects. To test this hypothesis, we looked for associations between disease severity (APACHE II and SOFA scores), death, haemolysis, inflammation and HO-1 (Fig. 4).

There was no association between APACHE II score at admission and any of the markers of haemolysis (Fig. 4A–D). However, APACHE II score was significantly positively associated with both IL-10 and HO-1 concentrations (Fig. 4E,F). Further, both IL-10 and HO-1 concentrations were both moderately, but statistically significantly, positively correlated with SOFA (Sequential Organ Failure Assessment) scores (IL10; r = 0.41, p = 0.0004, and HO-1; r = 0.38, p = 0.0009) (Fig. 4G,H). After adjusting for potential confounding, IL-10 was associated with APACHE II score but HO-1 was not (Table 1). Finally, we sought to characterize in-hospital mortality according to high or low (above or below median) concentrations of each analyte at admission. While neither anaemia nor HPX were significantly associated with mortality (Fig. S4, Table 2), high levels of plasma haem, IL-10 and HO-1 at admission were significantly associated with risk of dying (Fig. 4I–K, Table 2).

**Table 2:** Hazard ratios comparing mortality rates in different subgroups defined by immunological and clinical characteristics at admission.

Variables	Unadjusted^¶^HR (95% CI)	p-value	Adjusted^₡^HR (95% CI)	p-value
HO-1	4.93 (1.41,17.22)	0.013	4.05 (1.13,14.46)	0.031
IL-10	3.13 (1.01,9.65)	0.047		
Haem	4.04 (1.29,12.67)	0.016	3.34 (1.03,10.88)	0.045
Hb	0.70 (0.25,1.92)	0.483	0.75 (0.27,2.10)	0.588
HPX	0.53 (0.20,1.45)	0.219	0.62 (0.23,1.68)	0.348
APACHE II	3.23 (1.13,9.26)	0.029	2.64 (0.88,7.89)	0.083

^¶^Unadjusted hazard ratio (HR) comparing individuals above the median to those below the median, ^₡^Hazard ratio adjusted for log-transformed IL-10 response.

## Discussion

The key findings of this preliminary study are that; (1) the majority of sepsis patients are moderately anaemic at admission to ICU, (2) plasma concentrations of HO-1, the inducible isoform of haem oxygenase, are markedly raised in sepsis, (3) IL-10 concentrations, rather than haemolysis, correlate most closely with HO-1, and (4) high HO-1 and IL-10 concentrations, but not anaemia, at admission correlate with disease severity (APACHE II score) and mortality.

The primary function of HO-1 is to degrade haem, which is highly pro-oxidant and cytotoxic. Thus, HO-1 is tissue protective in inflammatory situations, reducing damage from free haem but also actively protecting tissues through the actions of the haem breakdown product carbon monoxide^15^. The essential role of HO-1 is illustrated by a case of human HO-1 deficiency which led to death in childhood accompanied by anaemia, intravascular haemolysis, leucocytosis and chronic inflammation^42^. Conversely, low-dose carbon monoxide therapy is beneficial in animal models of sepsis yet has failed in one study of experimental endotoxaemia in humans^43,44^. Importantly, while HO-1-mediated degradation of haem to carbon monoxide has been proposed as a major cell stress indicator^45^, the immunoregulatory cytokine IL-10 can also induce expression of the HO-1 gene, *HMOX-1*^29^. Similarly to IL-10^46^, HO-1 has also been linked to immune dysfunction and loss of resistance to infection^47^. These conflicting (tissue protective but immunosuppressive) actions of HO-1 and its differing modes of induction (haemolysis and inflammation) make it difficult to discern the risks and benefits of HO-1 induction during acute infection and to ensure an optimal balance between enhanced resilience and loss of resistance.

While anaemia, haemolysis and inflammation have all been described in sepsis patients^16,23,24,39,40,48,49^, the relationship between these processes and their collective relationship to disease severity is much less clearly established. Here, we sought to explore these parameters in a single patient cohort and to extend these analyses to their impact on the HO-1 pathway. Our patients had a median HPX that was markedly lower than the normal healthy range, consistent with depletion of plasma HPX due to scavenging of haem-haemopexin complexes. Three previous studies have found that patients who did not survive sepsis had lower levels of HPX than survivors^16,24,48^. Here, however, we compared survival in those with low and high HPX and found no association (Fig. S4B). In our cohort, those with the lowest HPX concentrations had the highest concentrations of HO-1 (consistent with haemolysis leading to liberation of free haem and induction of HO-1 and scavenging of haem-haemopexin complexes) but these patients were not necessarily the most anaemic. This may reflect that these patients are iron sufficient and able to compensate for modest levels of haemolysis by *de novo* red blood cell production. While high haem concentrations were associated by with reduced survival in hospital, low HPX at admission did not (Fig. S4).

Our data suggest that, in sepsis, maintenance of homeostasis in the face of overwhelming inflammation^49^ is the primary driver of the HO-1 pathway and that the hemolysis pathway of HO-1 induction is less important. Although the majority of patients in this study were anaemic, haemolysis may not be the sole cause of the anaemia. Anaemia of chronic disease (as suggested by high serum IL-6) may also contribute to the moderate anaemia seen in our cohort; hepcidin measurements would help to clarify this. Nevertheless, HO-1 concentration was moderately and significantly correlated with plasma IL-10 concentration and IL-10, in turn, reflected high concentrations of circulating inflammatory cytokines.

**Figure 5:**
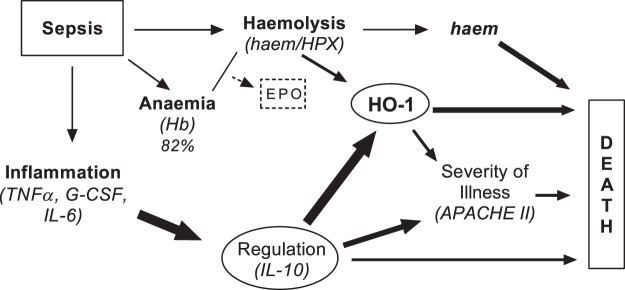
Proposed model of the relation between haemolysis, IL-10, HO-1 and mortality at admission of sepsis. The majority of sepsis patients at admission are moderately anaemic (low haemoglobin); however, those with the highest erythrocyte counts show elevated markers of haemolysis (haem and haemopexin, HPX) and elevated EPO. Further, systemic infection leads to robust inflammation and correlated with haemolysis (depleted haemopexin levels). Next, we observed that both IL-10 and haemolysis (depleted haemopexin) leads to the induction of haem-oxygenase 1 (HO-1). After partial correlation analysis for HO-1 induction which adjusted for either factor (IL-10 or haem and HPX), we found that IL-10 was the main driver of HO-1. IL-10 and HO-1 both individually showed a significant and moderate correlations to clinical severity score (APACE II, SOFA). Finally, high levels of IL-10, HO-1, and haem (i.e., above median value) at admission were positively associated with in-hospital mortality. Of note, this proposed model is limited to the ICU admission parameters measured in this study – long-term interaction pathways and their full consequences is currently unclear. The causal model (i.e., the direction of the arrows) was determined a priori and tested using the study data, Arrow size: 1 pt (p = 0.05–0.02), 3 pt (p = 0.019–0.001), and 6 pt (p < 0.001).

Despite the limitation that measurements were made only on the day of admission, the most parsimonious interpretation of these data (Fig. 5) is that inflammation drives a homeostatic regulatory response (mediated by IL-10), that this in turn induces transcription and translation of HO-1, and that HO-1 is the most proximal driver (of the parameters we have measured) of death in hospital. Haemolysis may exacerbate or prolong this response. However, this survival analysis does not account for other potential confounders, changes in the measured parameters over time, or readmission after discharge. One hypothesis to be explored in future, longitudinal and multicentre studies is that HO-1-mediated impaired resistance to infection^19^, either linked to or independent of IL-10, could be a significant cause of mortality in sepsis. Further, bacterial infection, *per se*, may also induce HO-1, as lipopolysaccharide can induce HO-1 via the transcription factor Nrf2^50^.

Understanding the cellular sources of HO-1 and the mechanisms by which it impairs immune function will also be important. Neutrophils are key to the immune defence against invasive bacteria. During sepsis, delayed neutrophil apoptosis^51^ accompanied by increased release of neutrophils into circulation^52^ results in neutrophilia. Nevertheless, neutrophil function is markedly diminished due to premature release of immature neutrophils from bone marrow^52^; these immature cells migrate sub-optimally in response to cytokines and nitric oxide and have reduced capacity to produce reactive oxygen species (impaired oxidative burst)^53–55^. This release of defective neutrophils from bone marrow is mediated in large part by the HO-1 pathway^19,56^. Although the underlying defect in neutrophil maturation has not been characterized in sepsis, we speculate that HO-1 (or the products of HO-1 enzymatic activity) may impact innate immunity. Further, if similar, persistent, HO-1-mediated neutrophil dysfunction occurs in bacterial sepsis, this may begin to explain the increased risk of recurrent infection in sepsis survivors^57–59^: up to 63% of sepsis survivors experience at least one subsequent episode of invasive bacterial disease within 12 months and this is fatal in 16.1% of cases^59^.

Further work is needed to assess the causal link, if any, between inflammation, IL-10, HO-1 and neutrophil dysfunction in sepsis. Implicating this pathway in the poor prognosis of sepsis patients offers options for improved management of sepsis patients and survivors. In the acute phase of disease, constraining the enzymatic activity of HO-1 by administration of the competitive inhibitor tin protoporphyrin IX (SnPP)^60^ may ameliorate the deleterious impacts on neutrophil function whereas in the convalescent phase, neutrophil respiratory burst assays may identify patients at particular risk of relapse or reinfection.

In conclusion, HO-1 is markedly elevated among sepsis patients. Interestingly, although many sepsis patients displayed evidence of haemolysis, IL-10 (as a marker of regulation of inflammation) appeared to be the main driver of HO-1 induction. High levels of HO-1 and IL-10 at admission were predictors of disease severity and mortality. Given the known impact of IL-10 and HO-1 on diminished phagocyte function, the role of HO-1 in sepsis warrants further investigation.

## Methods

### Ethical Statement and Sample information

The study was approved by the ethics committees of the London School of Hygiene and Tropical Medicine (reference number 11936) and Guys & St. Thomas’ NHS Foundation Trust (16/NI/0179). Informed consent was obtained from patients or, where they lacked competency, from personal legal representatives. Retrospective consent was sought from patients after they regained mental competency. Clinical data, including the Acute Physiology, Age and Chronic Health Evaluation (APACHE) II score^61^ and blood samples were collected from 70 sepsis patients on the day of admission to the intensive care units of Guy’s & St. Thomas’ Hospitals (London, UK) in accordance with the NHS guidelines and regulations. Complete blood counts (CBC) were performed using a DxH800 haematology analyser (Beckman Coulter). Plasma samples were aliquoted and stored at −80 °C until use. Identifying patient information was removed prior to sample distribution to the authors.

### Haematological definitions

Anaemia was defined using WHO guidelines for circulating levels of haemoglobin (g/dL)^28^. For males above 15 years of age these are: non-anaemic (>13), mild (11–12.9), moderate (8–10.9), and severe (<8) anaemia. For non-pregnant females above 15 years of age these are: non-anaemic (>12), mild (11–11.9), moderate (8–10.9), and severe (<8) anaemia. Haematological reference ranges were obtained from the hospitals’ clinical pathology service (ViaPath, London, UK) (Table S2).

### Plasma protein quantification

Enzyme-linked immunosorbent assays (ELISA) were conducted according to manufacturers’ instructions to measure plasma concentrations of haptoglobin (HPT, GWB-8DA44B, Genway Biotech), haemopexin (HPX, GWB-4B6D1A, Genway Biotech), haem oxygenase-1 (HO-1, ADI-EKS-800, Enzo Life Sciences), and erythropoietin (EPO, 442907, Biolegend Inc.). Colorimetric determination of haem in plasma samples was conducted according to manufacturers’ instructions (DIHM-250, Bioassay Systems). Samples were diluted prior to testing as follows: 1:50,000 (HPT); 1:100 (haem), 1:40,000 (HPX), 1:5 (HO-1 and EPO). Plasma concentrations of IL-6, TNFα, G-CSF and IL-10 were determined by magnetic bead multiplex assay (HCYTOMAG-60K, Millipore, UK) following the manufacturer’s instructions, and analysed on a Luminex 100 (LuminexCorp, Austin, USA) running Bioplex Manager software. Samples were not diluted for this assay. Seven patients had IL-6 concentrations above the software extrapolation range (>19 ng/mL). Samples giving values below the limit of detection were arbitrarily assigned a concentration at the limit of detection for the purposes of statistical analyses.

### Statistical analysis

Pearson’s correlation was used to identify statistical associations between markers of inflammation (IL-6, TNF-alpha, G-CSF, IL-10), markers of haemolysis (haem, HPX, HO-1) and measures of disease severity (APACHE II). In addition, we calculated partial correlations to adjust for confounding, after having selected potential confounders by applying the “back-door criterion”^62^ to the causal diagram in Fig. 5. Interpretation of correlation coefficients (r values) was as described by Ratner^63^; notably moderate (r = 0.3–0.7) and weak (r < 0.3). Kaplan–Meier curves and hazard ratios were used to investigate associations between mortality and clinical and immunological characteristics at admission. Analyses were conducted using GraphPad Prism 7 and Stata version 14.

### Data availability

The datasets used and/or analysed during the current study are available from the corresponding author on reasonable request.

## Electronic supplementary material

Dataset 1
