# Supplementary material for: Relationship between Anaemia, Haemolysis, Inflammation and Haem Oxygenase-1 at Admission with Sepsis: a pilot study
Source: Sci Rep. 2018 Jul 25;8:11198. doi: 10.1038/s41598-018-29558-5 (PMC6060141; doi:10.1038/s41598-018-29558-5)

**Supplementary Digital Content:**

**Relationship between Anaemia, Haemolysis, Inflammation and Haem Oxygenase-1 at Admission with Sepsis: a pilot study**

Ekregbesi, Phebe^1^; Shankar-Hari, Manu ^2^; Bottomley, Christian ^3^; Riley, Eleanor M. ^1,4^; Mooney, Jason P. ^1,4^

^1^ Department of Immunology and Infection, London School of Hygiene and Tropical Medicine, London, United Kingdom

^2^ Department of Critical Care Medicine, Guy’s and St Thomas’ NHS Foundation Trust, London, United Kingdom

^3^ Department of Infectious Disease Epidemiology, London School of Hygiene and Tropical Medicine, London, United Kingdom

^4^ Division of Infection and Immunity, The Roslin Institute, University of Edinburgh, Edinburgh, United Kingdom

Supplementary Figure Legends:

**Figure S1. Additional clinical readouts of infection during sepsis.** Clinical parameters upon admission to the intensive care unit (ICU) after sepsis diagnosis: **(A)** temperature, **(B)** platelet count, **(C)** lymphocyte count, **(D)** neutrophil count, **(E)** C-reactive protein (CRP), and **(F)** mean corpuscular volume (MCV). Dot plots show individual patient parameters. Black lines represent medians for septic patients. Black shaded areas represent National Health Service (UK) healthy reference ranges (see Supplementary Table S2). Sepsis patients, n =70.

**Figure S2. Additional cytokines and their relationship to haemolysis and HO-1 in sepsis patients.** Plasma concentration of **(A)** TNFα and **(B)** G-CSF for sepsis patients; n = 70. Dot plots show individual patient parameters. Black lines represent medians of sepsis patients. Correlations for the relationship of TNFα and G-CSF to **(C-D)** HPX, **(E-F)** HO-1, and **(G-H)** IL-10. Log-transformed data shown with linear regression line. Pearson r and p-value shown.

**Figure S3. Relation of inflammatory cytokines, anemia and heme.** Correlations for the relationship of hemoglobin **(A-D)** or haem **(E-H)** to IL-6, TNFα, G-CSF, and IL-10. Log-transformed data shown with linear regression line. Pearson r and p-value shown.

**Figure S4. Relation of inflammatory cytokines, anemia and heme.** Kaplan–Meier survival curves with hazard ratio comparing individuals above the median to those below the median for **(A)** haemoglobin (Hb) (median 10.9 g/dL), **(B)** haemopexin (HPX) (median 9.595 mg/dL), and **(C)** APACHE II score (median 18.5).

**Supplementary Tables:**

Table S1: Clinical characteristics of sepsis cohort.

| **Sepsis Cohort (n=70)** | | **N ( % )** |
| --- | --- | --- |
| Age (years) | Median (range) | 64 (18-89) |
|  | 18-65 | 36 (51.4) |
|  | >65 | 34 (48.6) |
| Sex | Male | 44 (62.9) |
|  | Female | 26 (37.1) |
| Site of Infection | Respiratory | 46 (65.7) |
|  | Wound & soft tissue | 9 (12.9) |
|  | Intra-abdominal | 8 (11.4) |
|  | Urosepsis | 6 (8.6) |
|  | Osteomyelitis | 1 (1.4) |
| APACHE II score *, median (range) | | 18.5 (8-37) |
| SOFA score †, median (range) | | 7.0 (3-16) |
| Mortality | | 19 (27.1) |

* APACHE II, acute physiology and chronic health evaluation II score. † SOFA, total sequential organ failure assessment score.

Table S2: Hospital Hematological Reference Values

| **Readout** | **Unit of Measure** | **Min** | **Max** |
| --- | --- | --- | --- |
| Temperature | celcius | 36.5 | 37.2 |
| Lymphocyte Count | x 10^3 cells /uL | 1.2 | 3.5 |
| Neutrophil Count | x 10^3 cells /uL | 1.5 | 7 |
| CRP | mg/L | - | <8 |
| Hematocrit - Male | % | 40 | 50.5 |
| Hematocrit - Female | % | 36 | 47 |
| RBC Count - Male | x 10^6 cells /uL | 4.4 | 5.8 |
| RBC Count - Female | x 10^6 cells /uL | 3.95 | 5.15 |
| Haemoglobin - Male | g/dL | 13 | 17 |
| Haemoglobin - Female | g/dL | 12 | 15 |
| Platelet Count | x 10^3 cells /uL | 150 | 400 |
| MCV | fL | 80 | 100 |
| Bilirubin | umol/L | - | <21 |

Data provided by NHS via ViaPath Pathology Services (London, UK)

**Supplementary Figures:**

Figure S1:


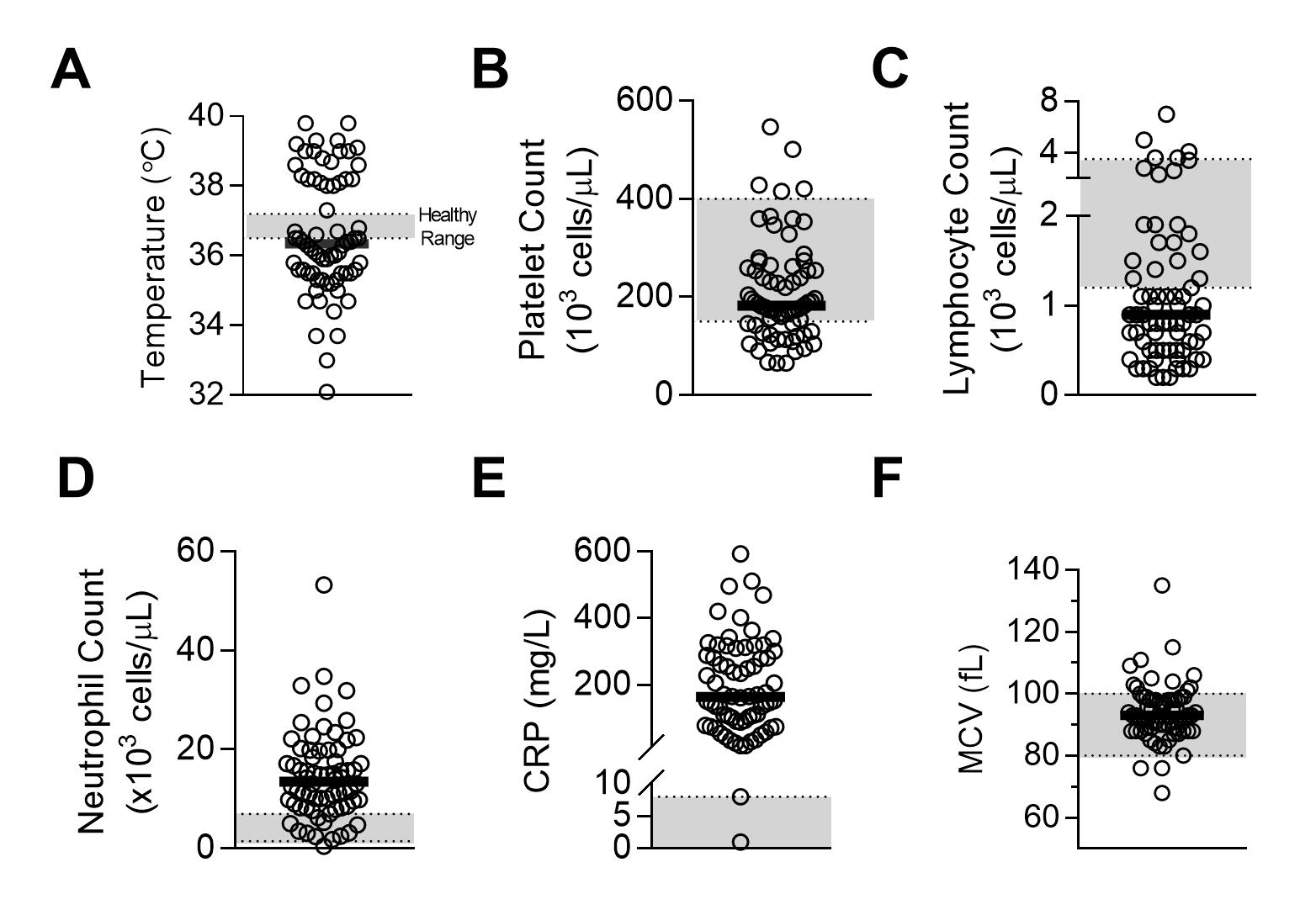


Figure S2:


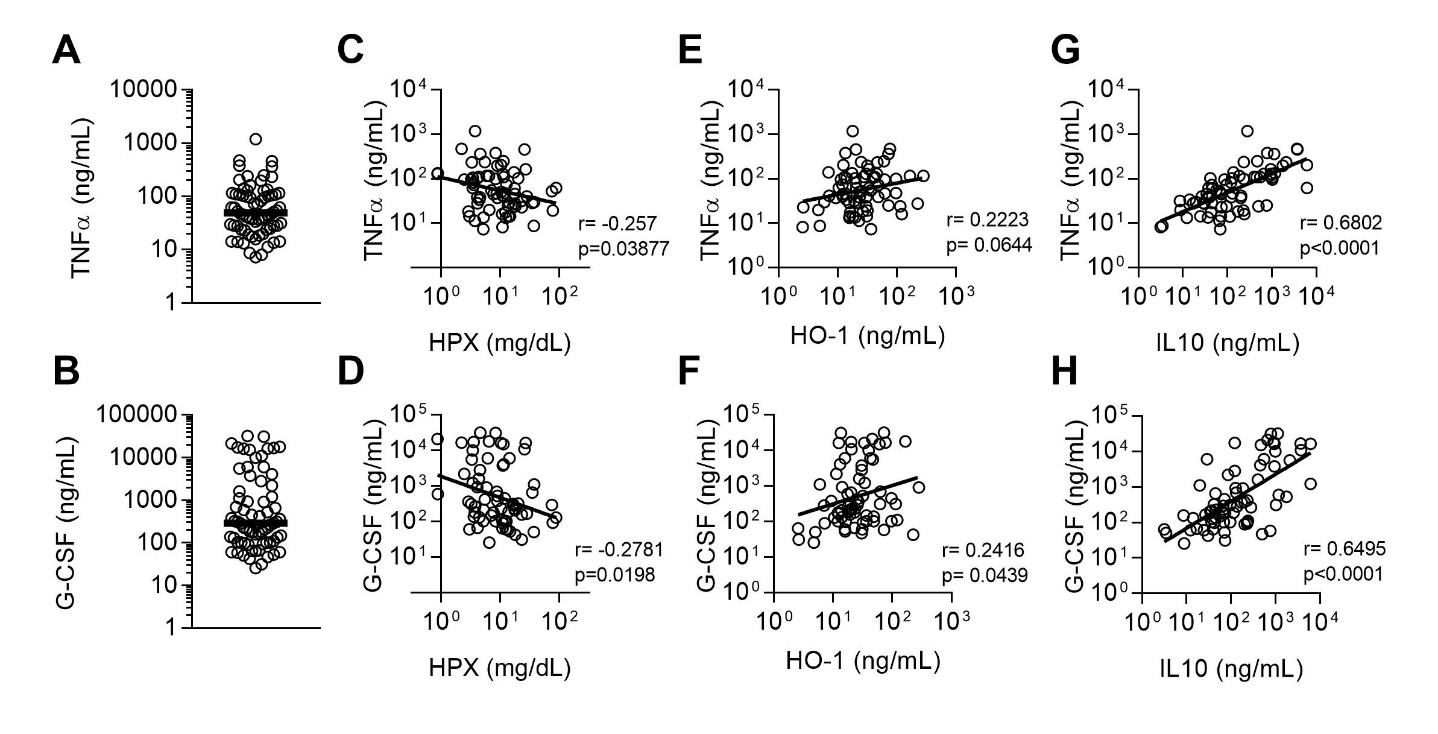


Figure S3:


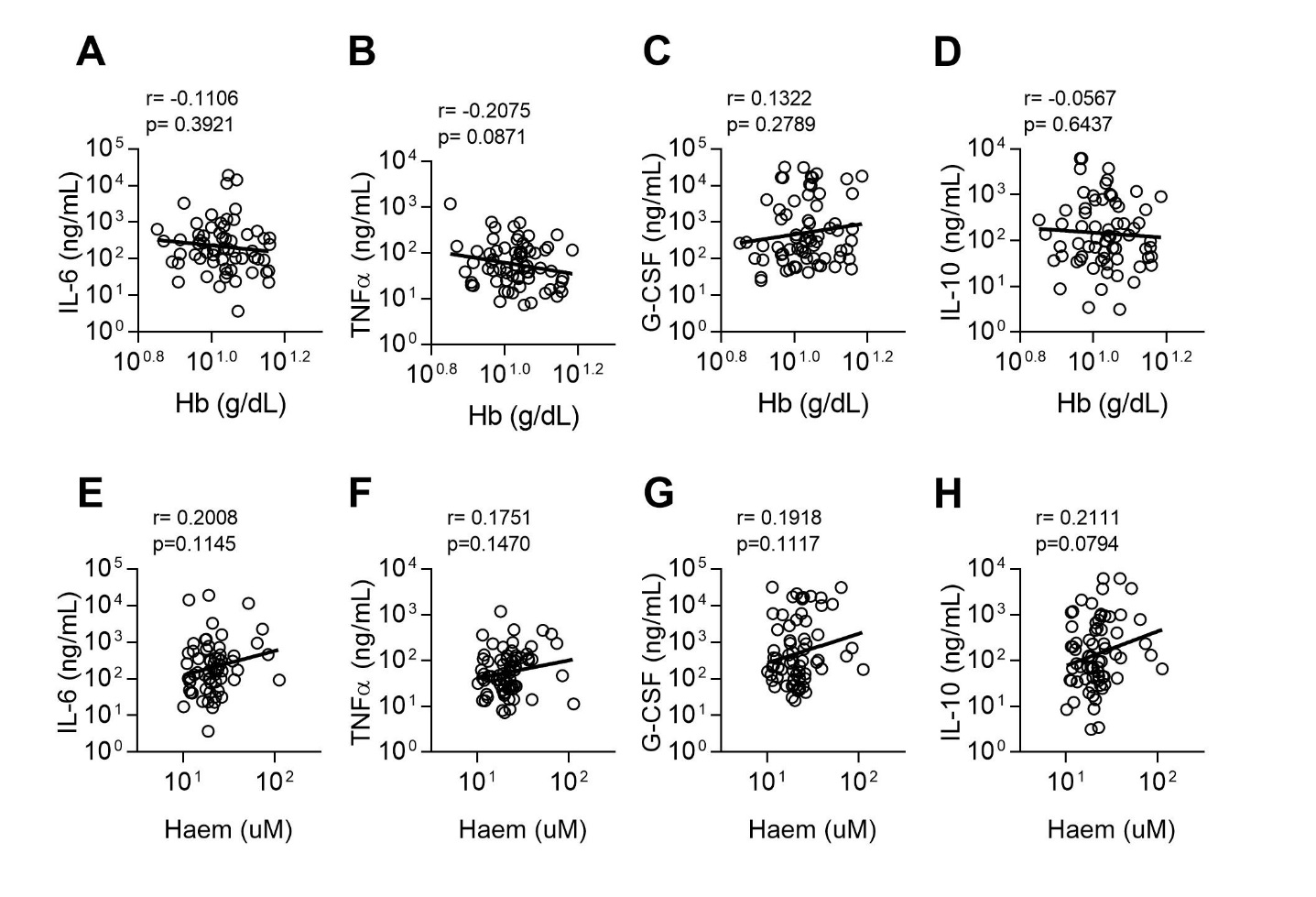


Figure S4:


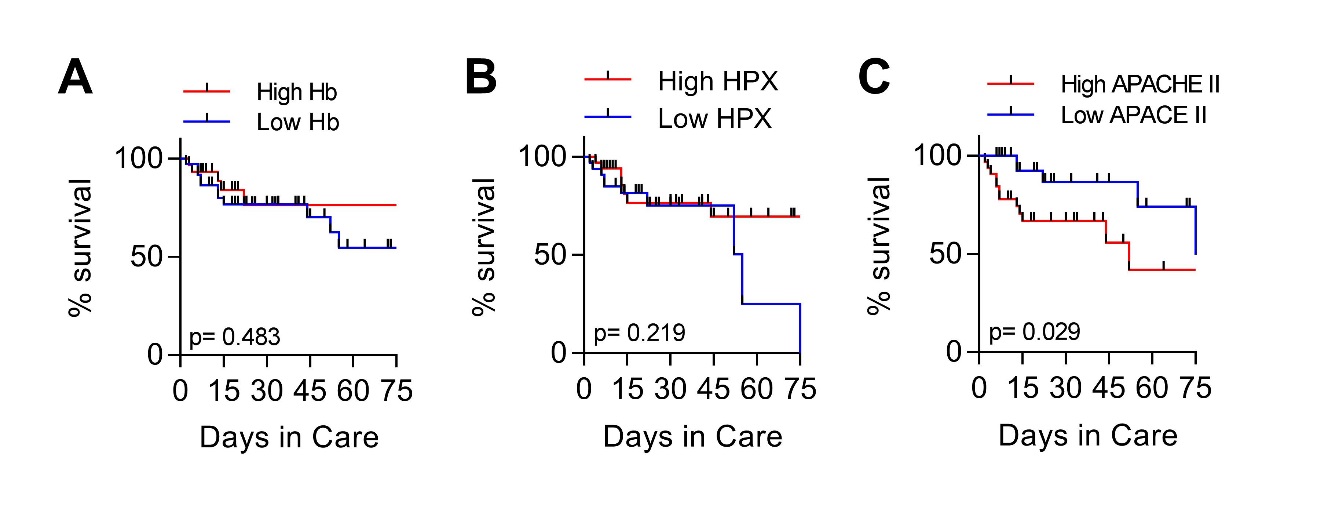

Supplement: Supplementary file 1 — Dataset 1 [file 41598_2018_29558_MOESM1_ESM.docx]
